# Hughes-Stovin syndrome: an unusual cause of pulmonary artery aneurysms

**DOI:** 10.1590/0100-3984.2015.0048

**Published:** 2016

**Authors:** Bruno Niemeyer de Freitas Ribeiro, Renato Niemeyer Ribeiro, Gláucia Zanetti, Edson Marchiori

**Affiliations:** 1 Instituto Estadual do Cérebro Paulo Niemeyer, Rio de Janeiro, RJ, Brazil.; 2 Universidade Federal do Rio de Janeiro (UFRJ), Rio de Janeiro, RJ, Brazil.

**Figure 1:**
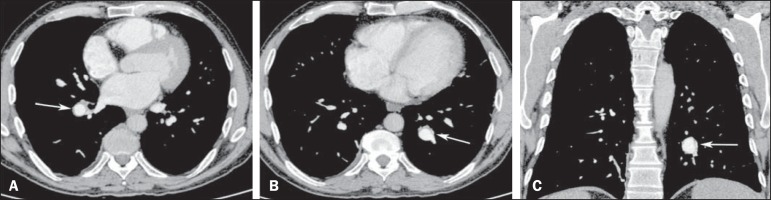
Contrast-enhanced computed tomography of the chest, with axial slices (**A,B**) and coronal slices (**C**), showing aneurysms in branches of the pulmonar arteries (arrows).

*Dear Editor*,

A 43-year-old male presented with a two-month history of persistent cough and fever, associated with recurrent episodes of superficial thrombophlebitis and venous thrombosis of the lower limbs. Physical examination revealed no evidence of oral or genital ulcers. Ancillary tests showed negative blood culture; no thrombophilia or neoplasia; negative serology; mild normocytic, normochromic anemia; elevated C-reactive protein; and elevated erythrocyte sedimentation rate. Contrast-enhanced computed tomography identified aneurysms in branches of the pulmonary arteries (Figure 1). The final diagnosis was Hughes-Stovin syndrome.

Idiopathic and vascular diseases of the thorax have been the subject of recent publications in the radiology literature of Brazil^(1-6)^. Hughes-Stovin syndrome is a rare condition, characterized by the combination of multiple pulmonary artery aneurysms and peripheral venous thrombosis, that mainly affects males (80-90% of cases) between the second and fourth decades of life^(7-11)^. Although the lesions often affect arteries and veins simultaneously (in 68% of cases), isolated arterial or venous impairments are reported at frequencies of 25% and 7%, respectively^(9,11)^.

In its typical presentation, Hughes-Stovin syndrome occurs in three stages^(7-9,11)^: in the first stage, there are signs and symptoms of thrombophlebitis; the second stage includes the formation and expansion of pulmonary artery aneurysms; and the third stage is characterized by aneurysmal rupture with massive hemoptysis, progressing to death. The formation of pulmonary aneurysms has been attributed to the weakening of the vessel walls by an inflammatory process. Other hypotheses proposed to explain these changes include septic embolism and angiodysplasia of the bronchial arteries^(8,9,11)^. Aneurysms can be single or multiple, unilateral or bilateral, and can even arise at other sites (in the iliac, femoral, popliteal, carotid, or hepatic arteries), although with a lower risk of rupture^(9-11)^.

Some authors consider Hughes-Stovin syndrome an incomplete form of Behcet's disease, due to the similarity between the two in terms of the clinical, radiological, and pathological findings^(7-11)^. Therefore, Behcet's disease, which typically affects young males, is the main differential diagnosis^(11)^. The major (and mandatory) criterion for a diagnosis of Behcet's disease is oral ulcers that recur at least three times within 12 months, which should be accompanied by at least two of the minor criteria (not necessarily simultaneously), including recurrent genital ulcers, ocular lesions, skin lesions, and a positive pathergy test^(12)^, none of which were observed in our patient. Other causes of pulmonary artery aneurysms are trauma, infection, pulmonary hypertension, and Marfan syndrome^(8-11)^.

There is no standard treatment for Hughes-Stovin syndrome, the most widely used treatment option being immunosuppression therapy involving a combination of glucocorticoids and cyclophosphamide, which has the potential to stabilize aneurysms or even promote regression in some cases^(11)^. The use of anticoagulants is controversial because of the risk of fatal hemoptysis, being allowed only in selected cases and provided jointly administered with immunosuppression therapy^(7-11)^. Other possible treatments include surgical resection and arterial embolization, which are used in most cases in which there is massive hemoptysis^(11)^.
